# Effect of the Extracts of the Spiderflower, *Cleome arabica*, on Feeding and Survival of Larvae of the Cotton Leafworm, *Spodoptera littoralis*

**DOI:** 10.1673/031.013.6101

**Published:** 2013-06-25

**Authors:** Afef Ladhari, Asma Laarif, Faten Omezzine, Rabiaa Haouala

**Affiliations:** 1 Department of Biology, Faculty of Sciences of Bizerte, Jarzouna 7021, Tunisia (UR03AGR04); 2 Laboratory of plant Protection, Regional Research Centre on Horticulture and Organic Agriculture (CRRHAB). Chott-Mariem 4042, Tunisia; 3 Department of Biological Sciences and Plant Protection, Higher Institute of Agronomy of Chott- Mariem, University of Sousse, 4042, Tunisia (UR03AGR04)

**Keywords:** antifeeding, bio-inesceticides, botanicals extracts, nutritional indices, toxicity

## Abstract

Aqueous and organic (hexane, chloroform, and methanol) extracts of siliquae, stems and leaves, and seeds of *Cleome arabica* L. (Brassicales: Capparidaceae) were evaluated in the laboratory for their antifeeding and insecticidal effect on larvae of the cotton leafworm, *Spodoptera littoralis* (Boisduval) (Lepidoptera, Noctuidae), using a leaf dipping bioassay with castor bean, *Ricinus communis* L. (Malpighiales: Euphorbiaceae), leaf discs. The polar extracts caused significant mortality. At the highest dose, *C. arabica* extracts exhibited significant antifeeding and phagostimulating activities against *S. littoralis* larvae. Under no-choice conditions, the methanol extract of siliquae was the most active, and the antifeedant index calculated over 24 hr for 3rd instar larvae varied significantly from 16 to 37%. Using nutritional indices, it was established that there was a significant decrease in growth rate concomitant with a reduction in consumption. These results suggest the presence of anti-feeding and/or toxic substances in the extracts that may be useful in developing bio-insecticides based on *C. arabica* extracts for use in integrated pest management of leafworm and other agricultural pests.

## Introduction

Pesticides are an important for maintaining a stable crop yield, but many of them are highly toxic and have long-term persistence in the environment. Despite all these efforts, losses due to these pests can annually reach 10–20% (Ferry et al. 2004) and still remain a challenge to be resolved (Zapata et al. 2009).

In recent years, attention has been directed towards using plant extracts to provide alternatives to synthetic insecticides. Plants have evolved many chemical defense mechanisms against insects (Wink 1993). As a result of interactions with insects, plants synthesize a broad range of different chemical compounds called secondary metabolites (Howe and Jander 2008), such as alkaloids, polyphenols, terpenoids, steroids, essential oils, lignans, sugars, and fatty acids, that protect the plants from insect pests (Regnault-Roger et al. 2004; Isman 2006) and are potentially suitable for use in integrated pest management (Schmutterer 1992). The majority of commercially produced botanical insecticides utilize the effects of plant metabolites that show acute or chronic toxicity to insects (Dayan et al. 2009; Pavela et al. 2009). Over 2000 species of plants are known to possess some insecticidal activity, by containing either antifeedant, repellent, or insecticidal compounds that enable the crude plant material, or an extracted active compound, to protect stored products (Klocke 1989; Bouda et al. 2001). Many compounds have been identified from numerous plant species, with the most promising ones for insect control coming from the families *Meliaceae, Rutaceae, Annonaceae,Asteraceae, Labiatae, Solanaceae*, and *Piperaceae* (Chaieb et al. 2004; Koul 2005; Chaieb et al. 2007).

Some species of Capparidacea possesses notable biological activities, such as antimicrobial (Mali 2010), anti-diabetic (Yaniv et al. 1987), analgesic, immune modulatory (Mali 2010), anti-inflammatory (Al-Said et al. 1988; Rossi et al. 1988), antioxidant (Germano et al. 2002), genotoxic (Sultan and Çelik 2009), anti-allergic, antihistaminic (Trombetta et al. 2005), antifungal (Ali-Shtayeh and Abu-Ghdeib 1999), antihepatotoxic (Gadgoli and Mishra 1999; Aghel et al. 2007; Mali 2010), and hypolipidemic activity (Eddouks et al. 2005). According to Willis (1966), *Cleome* (L.) is a large genus, with 150 species in the tropical and subtropical countries of both the Old and New World. The spiderflower, *Cleome arabica* L. (Brassicales: Capparidaceae), is widespread in North Africa. It has been used as folk medicine in the treatment of scabies and inflammation (Ahmad et al. 1990; Tsichritzis et al. 1993), rheumatic pains (Bouriche and Arnhold 2010), and as an antioxidant (Selloum et al. 1997). Yang and Tang (1988) reviewed plants used for pest insect control and found that there was a strong connection between medicinal and pesticidal plants, suggesting the possibility that *C. arabica* may also have useful insecticidal activity. Chemical compounds from *C. arabica* have been isolated from its aerial parts (Bouriche et al. 2003; Selloum et al. 2003; Bouriche and Arnhold 2010;). Some studies on Saudi Arabian *Cleome amblyocarpa* revealed the presence of 4 new dammarane triterpenes and 2 known compounds, cleocarpanol (Tsichritzis et al. 1993) and cabraleahydroxy lactone (Cascon and Browon 1972). An Egyptian study of this plant under the name *C. africana* described the presence of cleocarpanol and cabraleahydroxy lactone (Tsichritzis et al. 1993) together with stigma-4-en-3-one, lupeol, taraxasterol, and a membrane derivative (Jente et al. 1990). The leaves of *C. arabica* contain a number of glucosylated, rhamnosylated flavonols (Bouriche and Arnhold 2010).

The aim of this study was to evaluate the potential activity of the aqueous and organic extracts of Tunisian *C. arabica* species on feeding behavior and toxicity to larvae of the cotton leafworm, *Spodoptera littoralis* (Boisduval) (Lepidoptera: Noctuidae), a generalist herbivore that is a major pest on cotton and different horticultural crops (Gomez and Arroyo 1994). *S. littoralis* is continually active throughout the year and feeds on the leaves of more than 87 host plants belonging to 40 plant families, which makes it a model of a serious polyphagous pest (Sadek 2003).

## Materials and Methods

### Plant material

*C. arabica* was identified through the use of a Tunisian flora identification guide (Pottier-Alapetite 1979). A voucher specimen was collected in 2009 from the region of Gafsa (Tunisia), dried, and deposited in the herbarium of the High Institute of Agronomy of Chott Meriem, University of Sousse, Tunisia.

### Extract preparation

**Aqueous extracts.** Fresh *C. arabica* plants were rinsed with tap water and separated into siliquae, stems and leaves, and seeds Different organs were then oven-dried at 60° C for 72 hr and ground. Thirty grams of dried materials were extracted by soaking in 100 mL distilled water at ambient temperature for 24 hr in a shaker to give a concentration of 30% (w/v) dry tissue. The extracts were filtered several times and kept at 4° C in the dark until use.

Aqueous extracts were diluted with distilled water to give final concentrations of 2, 5, 10, 20, and 30% (w/v).

**Organic extracts.** Sequential extraction was carried out with organic solvents having increasing polarity: hexane, chloroform, and methanol. Fifty grams of dried powder of siliquae, stems and leaves, and seeds were immersed in the respective organic solvents for 7 days at room temperature. Organic extracts were evaporated to dryness under reduced pressure at 45–50° C using Rotavapour R-114 (Buchi, www.buchi.fr). Dry fractions were stored at 4° C until use. For testing, the residue was weighed and redissolved, in the same solvent, at concentrations of 0.1, 1, and 10 mg of residue/mL of solvent (100, 1,000, and 10,000 ppm) (Haouas et al. 2010).

Among organic extracts, Si had the highest yields with hexane and chloroform solvent. For the methanol, S extract had the highest yield (1.7%) followed by SL (1.33%) and Si (0.48%) extracts (Table 1).

**Table 1.**
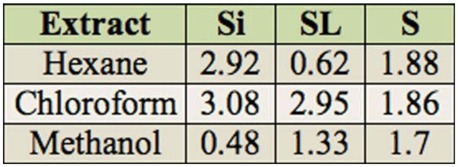
Yields, in percent of dry matter, of organic extracts of *Cleome arabica* siliquae (Si), stems and leaves (SL), and seeds (S).

### Insect rearing

Insects were obtained from a culture of *S. littoralis* maintained and reared on castor leaves, *Ricinus communis* L. (Malpighiales: Euphorbiaceae), in a climatic chamber at 25 ± 2° C, 75 ± 5% RH, and a 16:8 L:D photoperiod (El-Defrawi et al. 1964), and adults were provided with a 15% honey water solution.

### Effects of *C. arabica* extracts on larval survival

A leaf dipping bioassay method was adapted to evaluate insecticidal activity of different parts of *C. arabica* against the 3rd instar larvae of *S. littoralis*. The efficacy of the extracts were evaluated at the concentrations indicated above for aqueous and organic extracts. Leaf discs (5 cm in diameter) were prepared from castor bean leaves using a cork borer. Each disc was dipped in an extract for about 1 min. Control leaf discs were immersed in distilled water or in the same solvent and then dried at room temperature. After air-drying, each disk was placed in a clean Petri dish (1.5–9 cm). Ten 3rd instar larvae were starved for 2 hr and then released into the Petri dish. All bioassays were replicated 3 times. Larval mortality was recorded periodically during the bioassay (7 days). Percentage mortality was calculated using the following formula: Mc = (Mo — Me) / (100 - Me) × 100, where Mo = mortality rate of treated insects (%); Me = mortality rate of control (%); Mc = corrected mortality rate (%). Larvae were considered dead if they did not move when prodded with fine brush. Insect mortality was recorded in the end of experiment and adjusted for control using Abbott's correction (Abbott 1925).

### Effects of *C. arabica* extract on food consumption and utilization

The effects of aqueous and organic extracts of various parts *C. arabica* on food consumption and utilization by 3rd instar larvae were investigated using larvae reared on control diet after the second molt took place (< 24 hr). They were weighed and individually placed in Petri dishes. Then, they were fed with known weights of diets containing 0 and 30% (w/v)for aqueous extract and 10,000 ppm for organic extracts (n = 30 for each concentration) and allowed to feed for 2 days, a period slightly shorter than instar duration. At the end of experiment, larvae and feces were weighed, and food consumption was determined. The nutritional indices were calculated as follows:

Relative consumption rate: RCR = I / BaT

Relative growth rate: RGR = δB / BaT,

Approximate digestibility: AD = [(I - F) / I] × 100

Efficiency of conversion of ingested food: ECI = (δB / I) × 100

Efficiency of conversion of digested food: ECD = [δB / (I - F)] × 100

Metabolic cost: MC = 100 - ECD

Where: I = weight of consumed food; Ba = arithmetic mean of insect weight during the experiment = [(PF - PI) / log (PF / PI)]; PF = caterpillars final weight (mg); PI = caterpillars starting weight (mg); T = feeding period in days; δB = change in body weight; F = weight of feces produced during the feeding period (Waldbauer 1968; Farrar et al. 1989).

### Feeding assay with leaf discs

The antifeedant activity of the *C. arabica* extracts against 3rd instar *S. littoralis* larvae was investigated uisng a no-choice test because its design most closely approaches a practical application (Koul 2005). The feeding deterrence of SL, Si, and S extracts were evaluated at the 30% (w/v) and 10,000 ppm for aqueous and organic extracts respectively. Fresh castor bean leaf discs of 5 cm in diameter were punched using a cork borer and dipped in the corresponding test solution for around 1 min. Control leaf discs were immersed in distilled water or in the same solvent and let to dry at room temperature. Leaf discs and the 3rd instar larvae were introduced into each Petri dish (1.5–9 cm). The larvae were deprived of food for 4 hr before being placed individually in Petri dishes. Progressive consumption of leaf area by the treated and control larvae was recorded after 24 hr by laying the leaf on a graph paper and counting the number of 1 mm squares consumed. Meanwhile, a group of 30 arenas with 1 larva and 1 control disc in each was set up for control. The feeding deterrence (FD %) was calculated using the formula of Isman et al. (1990):

FD % = (C - T) / (C + T) × 100

T and C are leaf area consumed in the treated and control respectively.

### Statistical analysis

The laboratory bioassays were conducted in a completely randomized design with 3 replications. ANOVA and a post hoc Duncan test were performed with PASW Statistics 18 for Windows program (www.spss.com) to analyze treatment differences. The means were separated on the basis of least significant differences at the 0.05 probability level.

## Results

### Larval mortality under continuous exposure of *S. littoralis* to *C. arabica* extracts

The statistical analyses indicated significant differences in mortality in response to higher concentrations of aquous extracts of siliquae and seeds, after 7 days of treatment. The aqueous extract of seeds was the most toxic (Table 2).

Exposure to organic extracts resulted in decreasing larval survival in a dose- and time-dependent manner. Moreover, an increase in mortality rate with polarity was recorded (Table 3); methanol fractions of the 3 plant organs induced larvae mortality at the lowest concentration and application period. The siliquae methanolic extract was the most toxic, followed by seeds and then stems and leaves. At the highest concentration and 7 days after exposure, they induced 80, 63.3 and 56.6% mortality respectively. Chloroform and hexane extracts were less toxic than methanolic extracts. Chloroform yielded more toxic extract of siliquae and stems and leaves than hexane, while hexane yielded a more toxic extract of seeds than chloroform (Table 3).

**Table 2.**
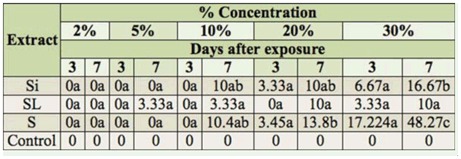
Percent mortality of *Spodoptera littoralis* in 3rd larval stage 3 and 7 days after exposure to different concentrations of *Cleome arabica* siliquae (Si), stems and leaves (SL), and seeds (S) aqueous extracts. Means with the same letters in a row are not significantly different at *p* < 0.05.

**Table 3.**
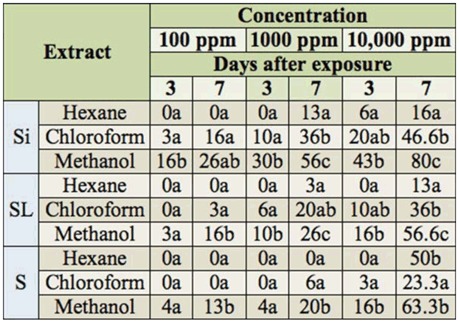
Percent mortality of *Spodoptera littoralis* in the 3rd larval stage 3 and 7days after exposure to different concentrations (100, 1,000, and 10,000 ppm) of organic extracts of *Cleome arabica* siliquae (Si), stems and leaves (SL), and seeds (S). Means with the same letters in a row are not significantly different at *p* < 0.05.

### Food consumption and utilization

Aqueous extracts influenced all nutritional indices (RCR, RGR, ECI, ECD, AD and MC) of the 3rd instar *S. littoralis* (Table 4). A significant reduction in AD, ECI, ECD, and RGR was observed, with an increase in MC in all tested aqueous extracts. RCR was reduced compared to the control when larvae fed on leaf disc treated with stems and leaves aqueous extract, while a slight increase was observed for siliquae extract. In addition, the seed aqueous extract did not induce changes in RCR compared to the control (Table 4).

**Table 4.**
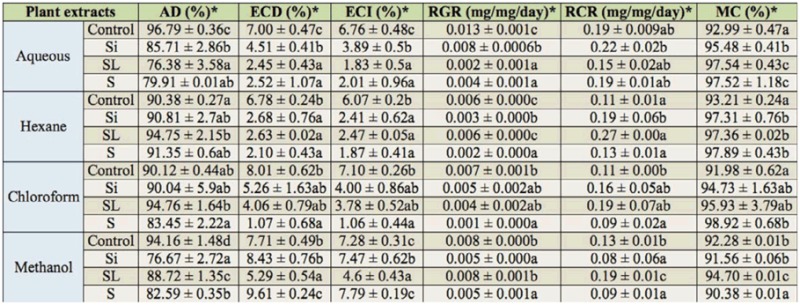
Nutritional indices of 3rd instar *Spodoptera littoralis* larvae fed for 2 days on treated fresh castor bean leaf discs by *Cleome arabica* aqueous extract (at 30% w/v) and organic extracts (hexane, chloroform, methanol) at 10,000 ppm of siliquae (Si), stems and leaves (SL), and seeds (S). Means with the same letters in a row are not significantly different at *p* < 0.05.

In order to segregate the effects of the extracts on behavior and physiology from toxicity, organic extracts were subjected to nutritional analysis (Table 4). Some nutritional indices of the 3rd instar larvae of *S. litorallis* were significantly different when fed different leaves with plant extracts. The relative growth rate (RGR) was significantly reduced after treatment with all aqueous extracts and some organic extracts. Larvae that fed on leaf discs treated with siliquae and seed methanol extracts exhibited a significant decreases in relative consumption rate (RCR). However, efficiency of conversion of ingested food (ECI) and the efficiency of conversion of digested food (ECD) significantly increased after treatment with seed and siliquae methanol extracts, and significantly decreased for the other treatments. The approximate digestibility (AD) was significantly reduced after treatment with methanol extracts.

### Antifeeding effect

**Figure 1.**
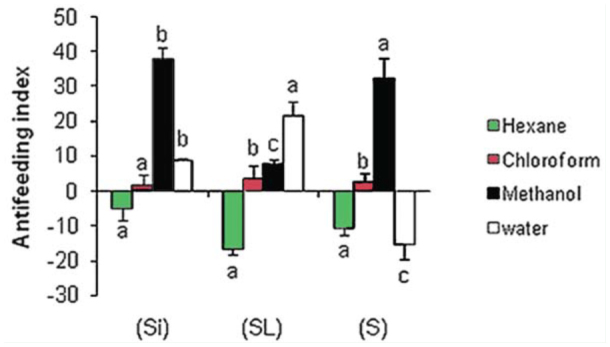
Antifeedant index of siliquae (Si), stems and leaves (SL), and seeds (S) aqueous extracts (at 30% w/v) and organic extract (at 10,000 ppm) of *Cleome arabica* against *Spodoptera littoralis* 3rd instar larvae in a no-choice test after 24 hours. The bars on each column show standard error. Different letters on columns indicate significant differences among extract treatments at *p* < 0.05. High quality figures are available online.

The antifeeding effect of *C. arabica* aqueous extracts at 30% (w/v) and organic extracts at 10,000 ppm were assessed on 3rd instar larvae of *S. littoralis* after 24 hr. Feeding deterrent and phagostimulant effects were both observed. For aqueous extract the highest significant antifeedant index was observed with stems and leaves extract (21.75 ± 3.95%) followed by siliquae (8.82 ± 0.44%), while seed aqueous extract showed a phagostimulating effect (-15.36 ± 4.35%) (Figure 1).

For organic solvents, the maximum antifeedant effect was recorded for the methanolic extract, with an index of 37.89% was obtained for siliquae, 32.15% for seed, and 7.8% for stems and leaves methanol extracts. Chloroform fractions exhibited no or poor antifeedant effect, with indices below 5%. In contrast, residue extracted by hexane showed phagostimulation effects for all extracts that ranged between -5.15 and -16.8% for the 3 organs (Figure 1).

## Discussion

The insecticidal activity of extracts of plant parts of *C. arabica* were evaluated against & *littoralis* by feeding 3^rd^ instar larvae leaf discs of castor, *R. communis* dipped in aqueous and organic extracts for 7 days. *Spodoptera littoralis* was chosen because it is a highly polyphagous insect (Brown and Dewhurst 1975; Holloway 1989) and is an economically important pest of cotton, vegetables, and ornamentals, and resistant populations cause severe problems in various regions of Tunisia.

The results showed that *C. arabica* extracts are endowed with biological activity, as 3rd instar *S. littoralis* larval survival fell in a dose dependent manner and time when they were fed on the plant extracts. It is possible that the decreasing survival was due to the antifeedant and toxic nature of the *C. arabica* extracts. No previous studies have been carried out on the effects of *C. arabica* extracts on *S. littoralis*, and no results are available on the plant's effect on other insects. *C. arabica* is classified as a medicinal plant that contains numerous biologically active compounds (Bouriche et al. 2003; Selloum et al. 2003). Some medicinal plants have been reported to show insecticidal properties and they have variously been used as crop protectants (Adedire and Lajide 1999; Ashamo and Odeyemi 2001; Omotoso 2004).

The toxic effects of *C. arabica* aqueous extracts were clearly observed at the highest concentration for seeds, followed by siliquae and then stems and leaves. Similar observations on other medicinal plant extracts' effects on several insects have been reported. For example, Oigiangbe et al. (2007) reported that extracts of *Alstonia boonei* reduced larval survival and weight in a dose dependent manner on *Sesamia calamistis* . Arora et al. (2011) found that extracts of *Withania somnifera* increased mortality of *Tribolium castenum* at the highest concentration. It is obvious that *C. arabica* aqueous extract contains several active constituents. Unlike synthetic insecticides, botanical insecticides contain mixtures of biologically active compounds whose biological effectiveness can be additionally increased by asynergic effects (Bhuiyan et al. 2001; Hummelbrunner and Isman 2001; Pavela 2008).

To determine the chemical group to which bioactive compounds of *C. arabica* belonged, a fractional extraction was conducted in 3 organic solvents with increasing polarity. The contrast between solvents indicates that the hexane extracts were significantly less potent than chloroform and methanol extracts; in addition, significant differences were detected between the latter two. Indeed, at 10,000 ppm, potent and moderate insecticidal activities were obtained respectively for siliquae methanol (80%) and chloroform (46.6%) extracts. This result indicates that, after extraction of molecules with the higher polarity solvent (methanol), *C. arabica* contains other bioactive molecules than can be extracted by chloroform that has medium polarity. However, Shadia et al. (2007) found that *C. ambylocarpa* methanol extracts induced only 50% mortality of *S. litorallis*. Therefore, the organic extracts must contain different types of bioactive molecules, as the difference would explain their differential effects. Moreover, the toxic post-ingestive effect of *C. arabica* extracts obtained in this study indicates that these organic extracts were toxic to *S. littoralis* larvae without killing the larvae at the beginning of the bioassay.

The specific toxic post-ingestive effect that *C. arabica* extracts caused to insects is not known. The results showed that *C. arabica* extracts reduced RGR rather than RCR in the post-treatment period. The RGR reached its lowest level (1%) after treatment with seed chloroform extract (Table 4). It may be inferred from the study that the decreased larval growth coupled with lower RGR, which is most likely due to longer retention of food in the gut for maximization of AD to meet the increased demand of nutrients (Senthil et al. 2005). The results revealed that aqueous and methanol extracts of *C. arabica* decreased the AD, while hexane and chloroform extracts did not induce any modification compared to control. Siliquae and seed methanol extracts induced a slight increase in ECI and ECD while, they provoked a decrease in RGR and RCR. A similar result was obtained for azadirachtin as its oral administration reduces RGR and RCR, but not ECI and ECD, which may be explained by its antifeedant activity (Koul and Isman 1991; Koul et al. 1996). In contrast, the other *C. arabica* extracts (aqueous, hexane, and chloroform extracts) induced a significant decrease in ECI and ECD values, which suggests that ingested *C.arabica* extracts also exhibited some chronic toxicity. In this respect, Abo El-Ghar (1985) recorded significant decrease in ECI values in fourth instar *S. littoralis* larvae when they were fed on castor bean leaves treated with petroleum ether extracts from tested plants, whereas feeding the larvae on ethanolic extracts resulted in an insignificant increase in food utilization. The variation in the nutritional indices of the 3rd instar larvae of *S. littoralis* could be due to the differences in the levels of allelochemicals in *C. arabica* different plant parts. According to Koul et al. (1990) and Appel and Martin (1992), one possible cause of the lower ECD for larvae is a reduction in the ability to detoxify an allelochemical occurring in the plant foliage or its extract, which consequently has a deleterious effect on the conversion of absorbed food to biomass. This may result from, for example, from direct interference of the allelochemical with some metabolic process (Slansky 1992) or indirect slowing of growth, thereby diverting a greater proportion of the absorbed food to respiration (Appel and Martin 1992).

The feeding behavior study showed that some aqueous and organic extracts of *C. arabica* had antifeeding effects with a marked differences, and their effectiveness against *S. littoralis* depended on the type of extracts. The present study clearly showed a slight antifeedant activity of stems and leaves (21.75%), followed by siliquae aqueous extract (8.82%), while seed aqueous extract induced phagostimulation (-15.36%). The residues obtained by organic solvent with low polarity showed phagostimulation effects, while the higher polarity extracts induced marked antifeedant effects. This result coincides with data reported by Houas et al. (2010), who mentioned a significant deterrent effect (78.55%) of methanol extracts of flowers of *C. segetum* at 10,000 ppm against *S. littoralis*, while a moderate antifeedant effect (45.23%) with *C. ambylocarpa* methanol extract was reported by Shadia et al. (2007). However, Zapata et al. (2009) found that for bioassays of antifeeding activity, extracts of stem bark of *Drimys winteri*, when extracted with organic solvents of low polarity, showed a large potential to interfere with the feeding of the generalist herbivorous *S. littoralis*. Thus, we inferred the antifeeding and phagostimulation effects of *C. arabica* extracts could be attributed to a mixture of toxic biomolecules possessing insecticidal properties present in the plant extracts.

The phytochemical investigation of aerial parts of *C. arabica* led to the isolation of phenolic compounds, alkaloids (Takhi et al. 2011), and a damarane triterpene (Khalafallah et al. 2009). Ismail et al. (2005) described known flavonol glycosides, such as 3-O-glucosyl-7-O-rhamnopyranosides, 3, 7-di-Orhamnopyranosides, and 3-O-glucopyranosides of quercetin, kaempferol, and isorhamnetin. Previous studies reported that isolated compounds such as alkaloids, coumarins, phenols, terpenes, and polyphenols have antifeedant and growth inhibiting effects on *S. littoralis* (Koul 2005; Pavela 2007; Susurluk et al. 2007). The responses of insects to these compounds vary greatly. For example, quercetin 3–0 rhamnosylglucoside is a phagostimulant to *Heliothis virescens*. Its influence on larvae of *Helicoverpa zea, H. armigera, Spodoptera littoralis, S. exiqua* and *S. exempta* depends on the concentration tested; at concentrations between 10^-4^ and 10^-5^ M it stimulated feeding, but at higher concentrations it was a deterrent (Blaney and Simmonds 1983). Alkaloids are found in large quantities in many plants and are used extensively as traditional insect repellents (Secoy and Smith 1983). Alkaloids affect acetylcholine receptors in the nervous system (e.g., nicotine) or membrane sodium channels of nerves (e.g., veratrin). Similar effects were induced by pyrethroids (i.e., monoterpene esters), which cause disturbances in the nervous system, leading to paralysis and mortality (Raffa and Priester 1985; Gershenzon and Croteau 1991). However, the action mode of these isolated compounds on *S. littoralis* is not known.

## Conclusion

In conclusion, the present results indicate that *C arabica* extracts possessed antifeedant and toxic effects on *S. littoralis* and inhibited growth through various metabolic processes. Therefore, these extracts could be incorporated during earlier instars, when they may be more toxic to larvae. Furthermore, the results suggest an interesting opportunity to develop bio-insecticides based on extracts from *C. arabica* for use in integrated pest management of insect pests that may affect crop production. Further study is in progress to isolate and identify the insecticidal constituents of this plant. Other areas requiring attention are the mode of insecticidal action and human safety issues, as well as the best formulation to improve insecticidal potency and stability and for cost reduction. Chemical studies of the methanolic extracts are currently in progress to identify the compound responsible of this behaviour perturbation on *S. littoralis*.

## Abbreviations

**AD**,: approximate digestibility
**ECD**,: efficiency of conversion of digested food
**ECI**,: efficiency of conversion of ingested food
**MC**,: metabolic cost
**RCR**,: relative consumption rate
**RGR**,: relative growth rate
**S**,: seeds
**Sl**,: siliquae
**SL**,: stems and leaves
